# Surface roughness and wear performance of Bioflx versus stainless-steel primary crowns (an in-vitro study)

**DOI:** 10.1186/s12903-025-05655-6

**Published:** 2025-03-05

**Authors:** Nancy Mohamed Metwally, Enas A. Elshenawy, Lamis Ahmed Elghareb

**Affiliations:** 1 Pediatric Dentistry Department, Faculty of Dentistry, Tanta University, Tanta, 31773 Egypt; 2 Dental Biomaterials Department, Faculty of Dentistry, Tanta University, Tanta, 31773 Egypt

**Keywords:** BioFLX crowns, Stainless-steel crowns, Pediatric crown, Two-body wear testing, Chewing simulation

## Abstract

**Background:**

Different kinds of crowns are used to restore primary teeth. Prefabricated crowns made of zirconia and stainless steel are frequently used. Bioflx crowns are a flexible and attractive substitute that combines the qualities of zirconia and stainless steel.

**Aim:**

This study aimed to compare BioFLX crowns to stainless-steel crowns regarding surface roughness and wear behavior.

**Methods:**

Two experimental groups based on the crown material (*N* = 14/group); group (1): stainless-steel crowns (SSC) and group (2): BioFLX crowns (FLX-C) were compared for surface roughness and wear resistance. Surface roughness was measured using an optical profilometer (white light interferometry, 20X objective lens). For wear measurement, seven primary molars for each group were prepared to receive crowns. Specimens were dynamically loaded (vertical loading, 50 N, 1.2 Hz) up to 100,000 cycles in a chewing simulator, and then wear volume was measured digitally using color mapping method. Data was analyzed using an independent T-test at a significant level of *P* < 0.05.

**Results:**

The FLX-C group had lower mean surface roughness (Ra)than the SSC group, however, the difference was not statistically significant according to the independent T-test (T = 0.704, *P* = 0.495). The FLX-C group experienced decreased wear volumes compared to the SSC group, with a significant difference between the two groups (T = 4.524, *P* = 0.001).

**Conclusions:**

Within the limitations of this study, it can be concluded that, in addition to their aesthetic superiority over SSC, BioFLX crowns have considerable wear resistance under several chewing cycles. Furthermore, their average surface roughness is comparable to that of SSC.

## Background

Early Childhood Caries (ECC) is the most common chronic disease in children and one of the main reasons for early deciduous tooth loss [1, 2]. Effective therapy is essential because tooth decay negatively impacts speech and masticatory function, as well as the proportions, stability, attractiveness, and quality of life of the arch [3, 4].

A primary tooth restoration needs to be aesthetically beautiful and strong, as it is expected to remain in the mouth until the primary tooth is removed. It also needs to be able to tolerate wear and tear [5]. The American Academy of Pediatric Dentistry (AAPD) recommends crown restoration for teeth with severe tissue loss, developmental imperfections pulpotomies, or pulpectomies [6]. Thus, it is possible to guarantee that the primary teeth’s mesiodistal dimension is retained, the tooth’s structural integrity is preserved, and its lifespan is extended [7]. Pediatric crowns should be simple to place, resistant to chewing stresses, avoid harming the opposing teeth, are biocompatible with surrounding tissues, and do not interfere with dental hygiene [4].

Stainless-steel crowns (SSC) are now the gold standard for restoring severely decayed primary molars [8]. The malleable alloy, along with thin-crown margins, allows for good adaptation and acceptable marginal quality [9].

Manufacturers introduce veneered stainless steel or complete zirconia crowns due to nickel content and unsightly appearance issues, but also face challenges like veneer breakage and invasive dental preparation during zirconia crown placement [10]. Moreover, the increased chewing forces of children with bruxism can cause the occlusal surface of the SSCs to get worn down and possibly perforated. The most frequent reason for SSC failure and occlusal surface perforation is occlusal wear [11]. Additionally, SSCs are exposed to oral environment for extended periods, influenced by physical and chemical factors such as abrasion, chewing, brushing, salivation, acidic beverages, and biofilm composition [12].

BioFLX crowns are a more recent advancement in pediatric dentistry that combine the qualities of zirconia and stainless steel crowns, making them renowned for their adaptability and flexibility [13]. These crowns are composed of a biocompatible hybrid resin polymer that addresses the ductility, color stability, and durability problems commonly associated with fiberglass-reinforced composite crowns. Surprisingly, BioFLX crowns are metal and bisphenol A-glycidyl methacrylate (Bis-GMA) free, guaranteeing a tooth-colored, metal-free restoration. It’s interesting to note that, similar to stainless steel crowns, BioFLX crowns offer a “flex fit” adaptation over the anatomic cervical convexity of primary teeth. However, like pediatric zirconia crowns, they also have the benefit of being more cosmetically acceptable and requiring less tooth preparation [14]. They also can withstand the highest loads [15] and exhibit more resistance to fracture than Zirconia crowns [16].

Since Bioflx is a novel material, there is a lack of research evaluating the laboratory mechanical and surface properties of Bioflx crowns and their impact on clinical outcomes [17]. Therefore, the primary objective of this in vitro study was to preclinically evaluate surface roughness and two-body wear volume after chewing simulation. The null hypothesis was that there would be no difference in terms of roughness and wear resistance between the chosen crown materials.

## Materials and methods

### Study design

**Fig. 1:**
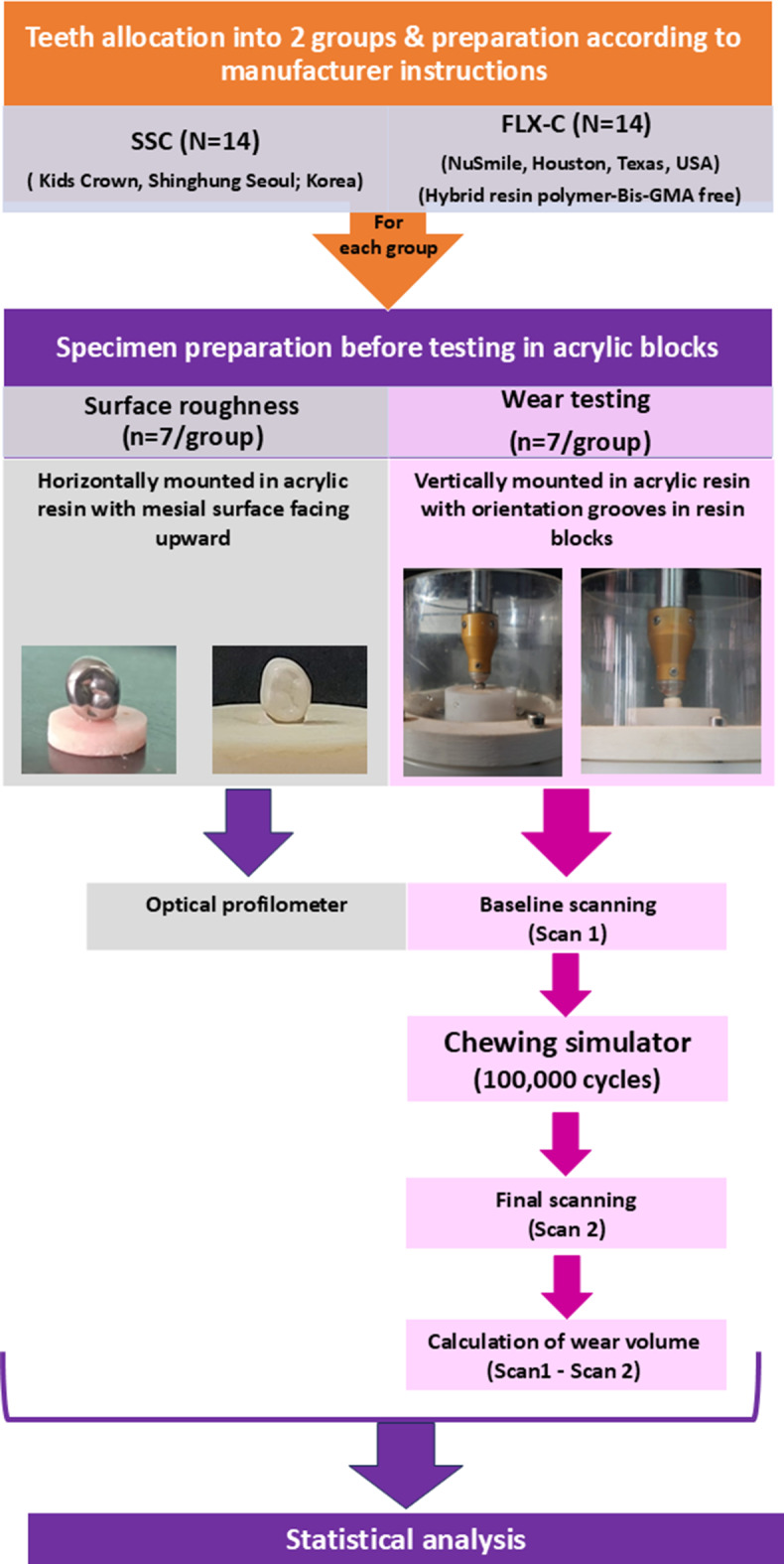
Flow chart demonstrating study design

A flow chart of the study design is presented in Fig. 1. This in vitro study included two different prefabricated pediatric crowns (stainless-steel crowns (SSC) (Kids Crown, Shinhung, Korea) and BioFLX crowns (FLX-C) (NuSmile Inc., Houston, Texas, USA) (*N* = 14/group).

Sample size calculation was performed based on the findings of an earlier study adopting 95% as the power of the study [18]. For wear assessment, a total of 14 molars, which were cemented to freshly extracted human primary mandibular first molars planned for serial extraction, were prepared to receive the crowns (*n* = 7/group). Primary first molars that were extracted and recommended for serial extraction were obtained (with the guardians’ informed consent, authorized by the Tanta University Faculty of Dentistry’s International Research Ethics Committee; file number: R-PED-10-23-3064). Regarding surface roughness and 2-body wear, a comparison of the two crown materials was made.

### Specimen preparation

Following the removal of any cavities present, a glass ionomer adhesive filling material (Medifil, Promedica, Germany) was placed for restoring the teeth. In order to ensure appropriate adaptation and adjustment, restorations were positioned as optimally as possible. Inadequately restored samples, badly destructed molars, or a tooth with compromised structure or cracks were not included in the study.

For SSCs, A pear-shaped bur was used to evenly reduce the occlusal surface by 1.0 to 1.5 mm after the preparation depth was standardized with 169 L guide grooves for occlusal depth. This results in the preservation of the original cusp contours [19]. Diamond burs with tapered ends were used for interproximal slicing both mesially and distally. FLX-Cs preparations were the same as the SSCs group based on the manufacturer recommendations. Standardized tooth preparation procedures had been performed on each tooth to prepare it for crown fitting. For both types of prefabricated crowns that were being tested (SSCs and FLX-Cs), a trial fit was performed before cementation, and the crown was chosen based on the mesiodistal breadth of the prepared teeth.

After tooth preparations, teeth were cleaned and dried for cement application. The crowns were cemented using a glass ionomer luting cement (Medicem, Promedica, GmbH). Specimens were stored in distilled water at 37◦C for 24 h before properties measurement.

### Roughness measurement

Specimens were horizontally mounted in acrylic resin (Acrostone cold cure special tray material, Cairo, Egypt) with the mesial surface upward, resulting in an almost ideally flat surface.

The evaluation of the surface roughness was carried out using a profilometer (ZYGO Maxim-GP 200, USA) over the mesial surface of the crowns. The surface was scanned using white light interferometry with a 20X Mirau objective lens and advanced texture analysis software giving all the surface-related data as follows: A Halogen lamp emits white light that strikes an optical cube beam splitter, which then directs the light downward towards an interferometric objective, either of the Michelson or Mirau type. This objective is regulated by a piezoelectric transducer (PZT) that facilitates phase stepping or scanning of white light fringes. The light reflected from the test samples returns through the interferometric objective, passes back through the cube beam splitter, and reaches the camera, resulting in the formation of interference fringes. By utilizing a computer equipped with sophisticated texture analysis software, comprehensive surface-related data can be acquired. This procedure was carried out on three separate sites, standardized to be in the center of the surface, away from the margins, and at an equal distance, and mean roughness average (Ra, µm) values were obtained for each specimen.

### Wear testing

Specimens, with the teeth aligned vertically, were resin mounted (Acrostone, Cairo, Egypt) and the outer surfaces of resin blocks were marked with three orientation grooves.

Specimens underwent a standardized dynamic loading method for 100,000 cycles by chewing simulator (Chewing simulator CS-4.4, SD Mechatronik GMBH, Germany) after being mounted opposing a vertical bar holding a 4 mm steatite sphere antagonist. Loading was in vertical direction starting at the functional cusp for 0.3 mm toward the central fossa. The dynamic loading settings were 50 N, 1.2 Hz, a loading speed of 20 mm/s, and a lifting speed of 60 mm/s, with continuous water rinsing [18].

The specimens were optically scanned using a 3D scanner (DOF—Freedom HD Dental Scanner) both before and after being subjected to chewing simulation (baseline and follow-up scans). Exocad^®^ DentalCAD, version 2.2 Valletta, Exocad GmbH, Darmstadt, Germany) was used to individually import the two scans of each specimen for analysis.

Then, the exocad program enabled a precise superimposition of each specimen’s baseline and follow-up 3D surface scans with the help of the three orientation groves on the sides of the resin block and non-abraded areas as a reference to analyze the quantity of wear. The color mapping method indicated substance loss, and mimics medical software (Mimics Innovation Suite 20, Materialise, Leuven, Belgium) was used to measure the volume of the wear facets and record it in cubic millimeters [20].

### Statistical analysis

Statistical evaluation was computed using SPSS 20.0 for Windows (SPSS Inc., IBM Company, USA). The normality of the distribution was determined by the Shapiro-Wilk test. Roughness and wear volume data, showing normal distribution, the difference between the two crown materials was compared statistically using an independent T-test. The significance level was set at *p* < 0.05.

## Results

**Fig. 2:**
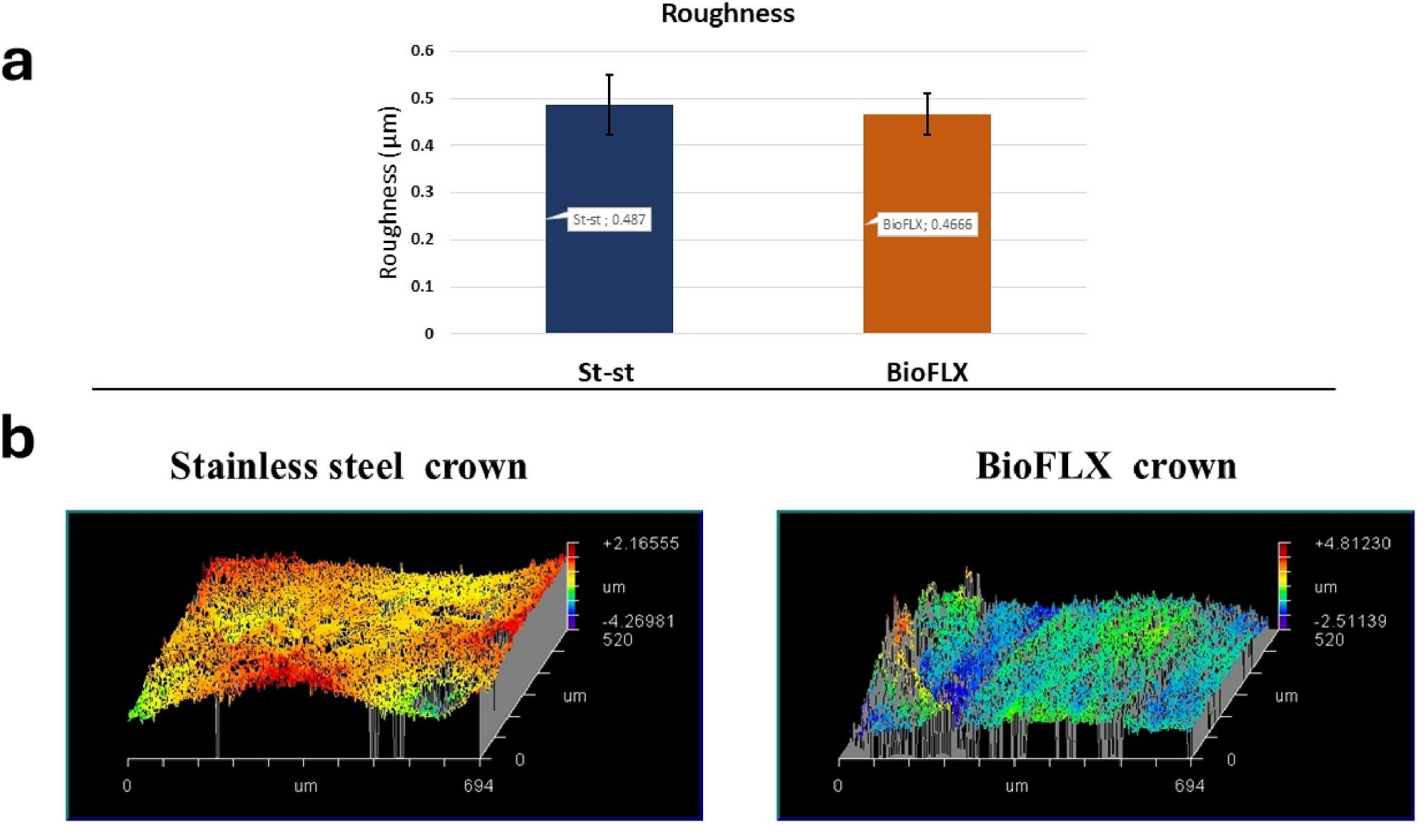
**a**, mean values of (Ra) (µm) for groups with standard deviation **b**, representative profilometer output of groups

Descriptive statistics for the roughness of both groups are presented in Fig. 2a. The mean surface roughness (Ra) of the FLX-C group was non-significantly lower than that of the SSC group according to the independent T-test (0.704, *P* = 0.495).

Figure 2b illustrates the surface topography of both groups as a result of profilometer output. There is some similarity in the value of scale between the two groups, however, the difference can be found in the direction of roughness, with the SSC group inclined toward elevations and the FLX-C group inclined toward depressions.

**Fig. 3:**
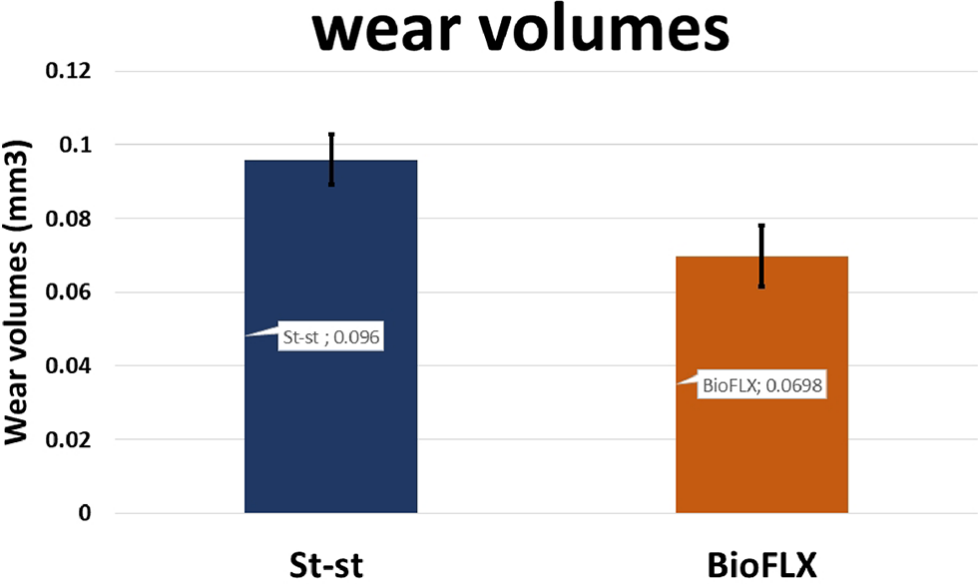
Mean values of wear volumes (mm^3^) for groups with a standard deviation

Figure 3 depicts the descriptive data of wear volumes of both groups. Here, wear volumes of the FLX-C group (0.069 ± 0.008) were significantly lower than that of the SSC group (0.096 ± 0.007) (T = 4.524, *P* = 0.001).

**Fig. 4:**
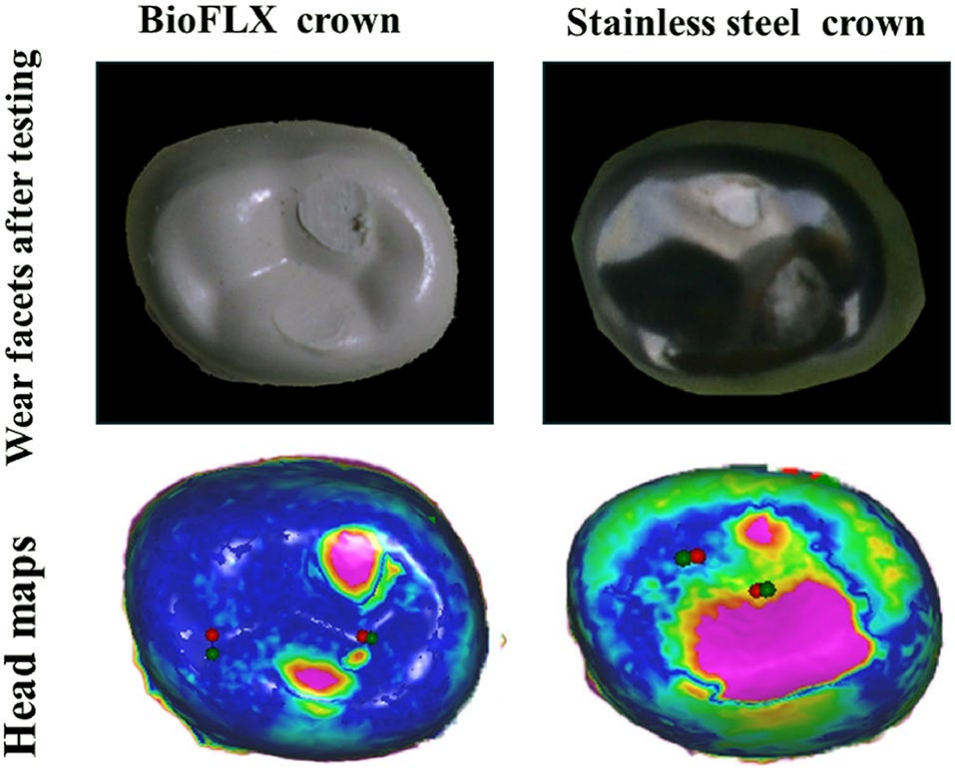
Representative photographs from groups, showing indentations produced by wear testing and the perforation observed in the SSC surface, and head maps of superimposed baseline and follow-up scans

The SSC’s worn surface indicated the development of an outer brownish oxide layer. One SSC showed a small perforation at the occlusal-lingual line angle at the end of 100,000 chewing cycles (Fig. 4). Wear traces for the FLX-C group revealed scratches pointing in the sliding antagonist’s direction together with tribolayers on the periphery of some wear facets (Fig. 4).

## Discussion

In vitro investigations are crucial for the preclinical evaluation of restorative biomaterials, so this study aimed to analyze roughness and wear resistance as biological and biomechanical features of crown materials, respectively, to provide timely predictions for clinical use.

Based on this study’s data, the null hypothesis was partially rejected, as there was no significant difference between the groups in terms of roughness, yet a significant difference in wear volumes was identified.

A smooth crown surface will improve oral hygiene by reducing the accumulation of residual food, minimizing plaque adhesion, and improving oral hygiene conditions [21, 22]. So, roughness measurement was undertaken to understand the difference between the two groups in terms of a tendency for plaque adhesion. Specimens were positioned horizontally with the mesial surface pointing upward in order to provide the largest flat area possible for testing.

The arithmetic average height, or Ra, is the most widely used roughness parameter. Considering that this parameter provides a good idea of height variations and is easy to define and calculate, it was chosen as the parameter used in this study to determine roughness [23].

Regarding wear measurement, a cycle number of 100,000 was determined, as it aligns closely with the typical in vivo period of six months to one year [24]. Wear in stainless steel crowns can be initially caused by plastic deformation, but with the repeated sliding force, actual wear is proceeded [18]. In our study, visual inspection of specimens was done to make sure that the facets produced were actual wear, and with inspection the surfaces showed loss of the metallic luster and thick margins of the facets, then quantitative measurement was done by exocad program.

Unlike our results, a clinical evaluation conducted by Patil et al. [25], found that after 12-month follow up, SSC performed better than in wear testing. However, their assessment method was different, by using Modified United States Public Health System criteria.

In this study, the surfaces of the FLX-C group showed significantly more endurance against chewing cycles compared to the SSCs group evident from less wear volumes. This can essentially be traced back to the material properties [26]. According to Kist et al., mechanical loads applied repeatedly to metals can cause fatigue cracks or cause the metallic surface to thin due to increased plastic deformation [12].

At the end of the chewing cycles, one SSC was perforated, while this did not happen with the FLX-C group. This is consistent with another study that found that the commercial SSC brand that we examined in this study had similar perforation between 80,000 and 120,000 cycles [27]. However, another investigation by Kessler et al. [18] found that these SSC defects occurred after a comparatively higher number of cycles (between 300,000 and 700,000), and they discovered that the cementation material affected wear volumes in SSCs. They also proposed the crown thickness as another reasonable factor [18]. In our instance, we believe that there may not have been enough cement support at the perforation site and occluso-lingual line angle.

In this investigation, scratches at wear facets have been observed in the FLX-C group. Similar scratches were detected, and Kessler and coworkers [18] indicated that the wear mechanism for polymeric crown material is a combination of abrasion and fatigue since the material’s surface may be acted upon by residual particles or the steatite antagonist, which could act as abrasives. Concurrent water rinsing in this study and thermocycling in theirs, however, would most likely remove these abrasives from the crown surface. Hereby, this water lubrication may have accounted for the formation of tribolayers at the periphery of the wear facet, according to Arieira et al., [28] the wear debris separated from the surfaces may have been compressed at the sliding interface, producing clustering and adhering of the material.

This study has certain limitations. Roughness was only evaluated under baseline conditions; the aging effect was not investigated. However, the primary goal was to examine this recently launched commercial product. Furthermore, in terms of wear, water was utilized to rinse rather than artificial saliva, which could have offered greater lubrication and clinical relevance. Even more, using water standardizes test conditions because saliva content differs among individuals. The wear of the antagonist was not assessed as well. In addition, hereby in the study, wear facets were observed on both on the buccal and lingual cusps, which may be a factor affecting the magnitude of load on both cusps. Therefore, for more standardization, it can be recommended to modify the steatite ball such that the load is focused on single cusp.

Further research is recommended to explore wear behavior over longer time frames within clinical studies. Additionally, evaluating more properties as marginal adaptation will offer a more comprehensive perspective into this new material. Moreover, assessing the gingival response to this material in clinical studies would be valuable.

## Conclusions

Within the limitations of this study, it can be concluded that, in addition to their aesthetic superiority over SSC, BioFLX crowns have considerable wear resistance under several chewing cycles. Furthermore, their average surface roughness is comparable to that of SSC.

## Data Availability

On reasonable request, the datasets utilized and/or analyzed during the present study are accessible from the corresponding author.

## Abbreviations

SSC: Stainless-steel crowns
FLX-C: BioFLX crowns
Ra: Mean surface roughness
ECC: Early Childhood Caries
AAPD: The American Academy of Pediatric Dentistry
